# Research Progress on Heavy Metals as Regulators of Bacterial Virulence in a *Caenorhabditis elegans* Infection Model

**DOI:** 10.3390/pathogens15030325

**Published:** 2026-03-18

**Authors:** Yiying Zhang, Xuanheng Tai, Kelan Wang, Ying Zhao, Xin Zhao, Wei Zou

**Affiliations:** 1 Yunnan Provincial Key Laboratory of Public Health and Biosafety, School of Public Health, Kunming Medical University, Kunming 650500, China; zyy77789@163.com (Y.Z.); ttll22007@163.com (X.T.); kelanwang11@163.com (K.W.); 2 Infection Control Office, Xi’an Public Health Center, Xi’an Emergency Medical Center, Xi’an 710200, China

**Keywords:** heavy metals, bacterial virulence, *Caenorhabditis elegans*, host–pathogen interactions, environmental signaling, risk assessment

## Abstract

Heavy metal pollution is increasingly recognized, not merely as a source of static toxicity, but also as a driver of dynamic microbial regulation. At sublethal concentrations, these pollutants function as critical environmental cues that reshape microbial evolutionary trajectories. This review elucidates how low-dose heavy metals bypass acute cellular damage to instead engage bacterial chemical-sensing networks, systematically upregulating virulence factors, biofilm architecture, and the co-selection of antibiotic resistance. By leveraging the *Caenorhabditis elegans* (*C. elegans*) infection model (a platform defined by its evolutionarily conserved innate immune architecture), we dissect the tripartite interplay between environmental metal flux, bacterial pathogenic output, and host immunological defense. We synthesize empirical evidence from the *C. elegans* model to highlight how heavy metals modulate bacterial virulence and host defense mechanisms, thereby providing new insights into the indirect health risks of environmental pollutants and their implications for redefining public health exposure thresholds and infectious disease control in the Anthropocene.

## 1. Introduction

Heavy metal pollution has evolved into a global issue transcending national borders, intertwining ecological security with public health. The combined drivers of industrialization, urbanization, and intensive agriculture have led to the simultaneous accumulation and spreading of heavy metals such as arsenic, cadmium, lead, copper, and zinc across soil, water, and sediment. Their non-biodegradable nature confers persistent chemical activity. Transmission through food webs amplifies effects at each trophic level, causing ecological risks to expand exponentially with increasing trophic levels. This imposes long-term, irreversible pressures on ecosystem structural stability and human health [1].

Traditional toxicology attributes the mechanism of high-dose heavy metal effects to three primary pathways: first, triggering oxidative stress to cause a sudden increase in intracellular ROS; second, covalently binding to protein sulfhydryl groups to cause enzyme inactivation; and third, inhibiting DNA repair systems, thereby inducing gene mutations and even carcinogenesis, ultimately causing observable pathological damage to multicellular organisms [2]. Within this framework, heavy metal hazards have long been simplified into a single dose response curve, shifting research focus toward acute poisoning treatment and immediate tissue lesions under direct exposure. However, real-world exposure scenarios more commonly involve low-dose, long-term continuous input, often at concentrations below acute thresholds. Within this sublethal or subinhibitory range, heavy metals undergo a fundamental biological role shift: they act not merely as cytotoxic agents but as critical environmental cues and simultaneously become selective pressures driving microbial ecological evolution [3]. To counter this stress, bacteria have evolved a multi-tiered chemical sensing-response network. This network activates classical detoxification and metal homeostasis modules while simultaneously feeding signals into virulence regulation. This manifests as multiple phenotypic outputs, including upregulation of virulence gene expression, enhanced biofilm synthesis, activation of quorum sensing systems, and adaptive rearrangement of central metabolic flux [4]. These findings extend the ecological and health risks of heavy metals from “static toxicity” to “dynamic regulation,” suggesting they can actively intervene in the real-time equilibrium of microbial behavior, interspecies interactions, and pathogen–host relationships. The resulting indirect health risks may surpass direct toxic effects in terms of concealment and scope. However, current environmental risk assessments remain centered on macroscopic indicators like mortality rates or morphological defects, systematically overlooking the complex chain of indirect effects low-dose heavy metals exert on host health through microbial pathways [5].

From an ecosystem perspective, chronic low-dose heavy metals have evolved into a constant stress driving microbial adaptive evolution. To maintain survival advantages, bacteria utilize metal ions as signaling scaffolds, constructing a unified regulatory network that simultaneously controls metal tolerance, antibiotic resistance activation, and virulence factor expression. This reveals a potential chain reaction: “environmental pollution, drug resistance transmission and virulence enhancement.” Resistance genes and metal tolerance genes often coexist within the same mobile genetic element. Metal ions synchronously regulate biofilm thickness, collective behavior, and host infection efficiency through quorum sensing and two-component systems, thereby conferring dual roles as both pollutants and core signaling factors [6].

To decipher the heavy metal pathogen host tripartite interaction, a living model that approximates physiological conditions while remaining amenable to manipulation is required. *Caenorhabditis elegans* (*C. elegans*), with its transparent body, short life cycle, complete genome, and ease of genetic manipulation, serves as an ideal platform. This model possesses innate immune signaling pathways highly conserved with mammals, including the PMK-1/p38 MAPK pathway for bacterial defense and the KGB-1/JNK-like MAPK pathway for environmental stress responses [7]. Through transgenic strains, fluorescent reporter systems, and infection survival assays, researchers can systematically reveal the dynamic equilibrium between heavy metal regulation of bacterial virulence and host immunity at the organismal level, elucidating the regulatory mechanisms of environmental pollution on infection progression. The *C. elegans* model, with its simple structure and conserved signaling pathways, serves as a bridge connecting environmental toxicology and host pathogen interactions. This model not only validates the functional roles of heavy metal signaling molecules in vivo but also reveals mechanisms by which pollutants indirectly exacerbate infection risks through the microbe host interface. Existing research provides evidence: when heavy metals enter the host, the expression levels of pathogen virulence proteins, the production of host antimicrobial peptides, and the release rhythms of signaling molecules undergo synchronized shifts. After the change curves of these three factors intersect, the infection process is modulated, leading to either shortened or extended host survival times [8]. The convergence of multiple factors elevates heavy metal pollution beyond traditional environmental science, making it a critical factor in public health and infectious disease control.

Drawing on domestic and international literature, this paper redefines heavy metals as bacterial virulence modulators. The review first outlines the full scope of research before focusing on the *Caenorhabditis elegans* infection model. Within this model, each step (how metals are sensed by bacteria, how virulence genes are activated, and how host immunity is weakened) is dissected into discrete experimental evidence points, with empirical evidence and phenotypic records provided for each. Building upon this foundation, the authors propose an integrated descriptive framework for the “environmental metal bacterial virulence host defense” axis, which systematically collates and summarizes the tripartite interaction mechanisms revealed by existing research.

## 2. The Dual Identity of Heavy Metals in Biological Systems

### 2.1. Environmental Distribution and Chemical Forms

**Figure 1:**
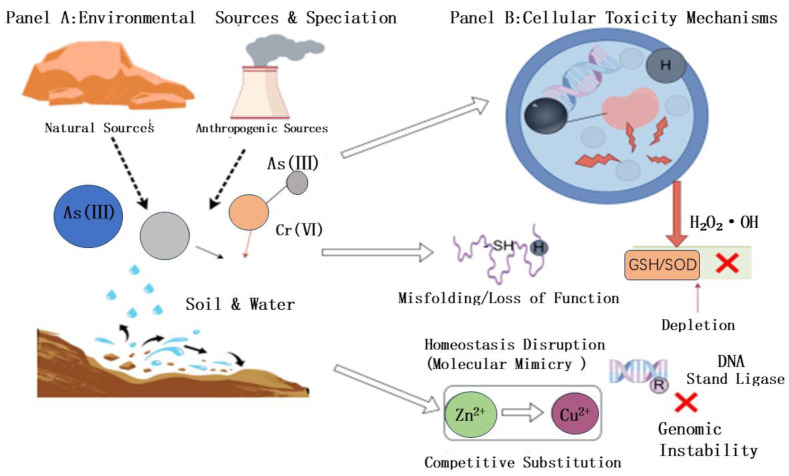
Environmental fate and multi-mechanistic toxicity of heavy metals in eukaryotic cells. Arrow and Symbol Key: **Dashed-line arrows**: Represent the release and transport pathways from natural and anthropogenic sources into the environment. **Solid black arrows**: Indicate chemical speciation and transformation within soil and water. **Large hollow arrows**: Denote the transition from environmental exposure to cellular uptake and toxicity processes. **Solid red/pink arrows**: Represent the induction of oxidative stress (ROS) and the subsequent depletion of antioxidant defenses (GSH/SOD). **Horizontal white arrow**: Illustrates competitive substitution (molecular mimicry) of essential ions (e.g., Zn^2+^) by toxic ions (e.g., Cu^2+^). **Red “X” symbols**: Signify the inhibition of enzymatic activities or the failure of DNA repair mechanisms, leading to genomic instability.

The quantitative assessment of heavy metal biological effects requires the establishment of a systematic description of their spatial distribution patterns and chemical transformation pathways. This report focuses on the dual-source characteristics (natural and anthropogenic inputs) of elements such as arsenic, cadmium, lead, copper, and zinc. Natural background levels primarily originate from rock weathering and geological cycling processes. Anthropogenic sources include mineral extraction, metal smelting, industrial wastewater discharge, field application of metal-containing pesticides and fertilizers, and improper disposal of electronic waste. Each of these activities shortens migration time constants while increasing surface enrichment fluxes (Figure 1 panel A).

Once various metal pollutants infiltrate and persist within complex environmental media such as soil, water bodies, or sediment deposits, simply measuring their cumulative total often fails to accurately assess their potential ecological hazards. This is because the trajectory of environmental behavior, the extent of actual uptake and utilization by organisms, and the severity of ultimate toxic effects are fundamentally controlled by the specific chemical speciation of metal elements within the medium. The so-called chemical form essentially encompasses the specific molecular structural forms, valence state distributions, and binding modes exhibited by metal elements under particular environmental biochemical conditions. It is precisely these microscopic structural differences that directly determine the solubility and diffusion capacity of metal ions in liquid environments, their reactivity in chemical processes, and their transport efficiency across biological membranes into organisms [9]. Taking the toxicological differences among inorganic arsenic compounds as an example, the reason trivalent arsenic (arsenite As(III)) exhibits far greater toxicity than pentavalent arsenic (arsenate As(V)) stems from its exceptionally high chemical affinity for the sulfhydryl functional groups within biological protein molecules. This allows trivalent arsenic to rapidly bind to key metabolic enzymes, irreversibly disrupting normal physiological and biochemical pathways, ultimately leading to enzyme inactivation and cellular dysfunction [10]. A similar situation applies to chromium. Hexavalent chromium (Cr(VI)), leveraging its potent redox potential and exceptional water solubility, exhibits a broader migration range and more pronounced biological toxicity in environmental media compared to trivalent chromium (Cr(III)).

In-depth investigations into the biological effects of heavy metals must be conducted within the core framework of specific environmental chemical speciation. This requires not only examining static speciation distributions but also comprehensively evaluating the complex dynamic transformations and ultimate fate of metal speciation driven by multiple environmental factors. These include alternating redox potentials, dynamic pH fluctuations, and evolving organic matter content.

### 2.2. Typical Toxicity Mechanisms in Eukaryotes

Heavy metals have long been defined as harmful agents with typical toxic effects within traditional toxicology because they can breach eukaryotic cell defense barriers and inflict irreversible structural damage and functional disruption at multiple microscopic levels. This includes compromising cell membrane integrity, damaging the active sites of key intracellular metabolic enzymes, and destabilizing core genetic material. These extensively validated classical cytotoxic pathways not only elucidate the direct biological hazards and lethal mechanisms of heavy metals but also provide indispensable theoretical reference points and comparative benchmarks for subsequent in-depth analysis of how microbial communities evolve specific adaptive mechanisms (through genetic mutation or horizontal transfer) under the sustained environmental selection pressure of heavy metals.

The induction of severe oxidative stress within biological cells by toxic heavy metals constitutes the core toxicological basis of their pathology. Typical pollutants such as cadmium ions (Cd^2+^) and trivalent arsenic (As^3+^) disrupt intracellular redox homeostasis through microscopic pathways, such as the Fenton reaction mechanism or physical interference with the normal operation of the mitochondrial electron transport chain catalyzing the explosive proliferation of reactive oxygen species (ROS) components, including superoxide anion (O_2_^−^), hydrogen peroxide (H_2_O_2_), and hydroxyl radicals (•OH), highly reactive oxygen species (ROS) with potent chemical destructive capabilities to proliferate explosively [11]. These high-energy oxidizing molecules then indiscriminately attack intracellular genetic material, membrane lipid systems, and functional proteins. They cause DNA strand breaks and base modifications while triggering lipid peroxidation, leading to membrane structural collapse and protein oxidative denaturation and inactivation. Beyond such direct oxidative damage, heavy metal elements can also deplete reserves of endogenous small-molecule antioxidants like glutathione (GSH) or specifically inhibit the active centers of key defense enzymes such as superoxide dismutase (SOD) and catalase (CAT) to simultaneously weaken the cell’s own antioxidant defense barrier. This forces the cellular organism into a metabolic imbalance where the rate of oxidative damage accumulation far exceeds repair capacity, ultimately irreversibly inducing comprehensive cellular dysfunction or initiating a programmed death cascade reaction [12].

Heavy metal ions, leveraging their exceptionally high chemical affinity for functional side-chain groups of biomolecular proteins (particularly sulfhydryl (-SH) groups) can bypass steric hindrance to directly bind and occupy the catalytic active sites of various key metabolic enzymes, thereby inducing irreversible functional inhibition. Typical toxins such as arsenic and form highly stable binding complexes with cysteine-rich enzymes like pyruvate dehydrogenase. This forcibly distorts the enzyme’s three-dimensional conformation, completely eliminating its biological activity while triggering comprehensive disruption of core physiological processes, including cellular energy metabolism networks [13]. Lead ions (Pb^2+^), sharing a similar pathological pathway, also specifically targets and blocks the biocatalytic activity of δ-aminolevulinic acid dehydratase (δ-ALAD) and ferrochelatase in the heme biosynthesis cascade. The resulting inhibition of heme synthesis constitutes the decisive molecular biological basis for the development of anemia and neurological toxicity observed in clinical pathology [13].

Toxic heavy metals produce molecular mimicry effects due to their high physicochemical similarity to essential ions such as zinc, calcium, and iron. By competitively occupying the binding sites of metalloenzymes and structural proteins, they disrupt trace element homeostasis. For example, cadmium (Cd^2+^) can not only displace zinc in zinc finger proteins, leading to loss of function in transcription factors and enzymes, but also block calcium signaling pathways. Even excessive accumulation of essential elements like copper and zinc can cause toxicity by displacing other functional metals at incorrect sites. Such disruption of metal homeostasis directly affects core cellular physiological processes such as gene expression and signal transduction [14].

The intestinal epithelial cells of *C. elegans* are not only its primary digestive organ but also the first line of biological defense against heavy-metal uptake and pathogen colonization. This transparent tissue is functionally highly analogous to the human intestinal mucosal barrier and is responsible for initiating the earliest defensive responses. When heavy metals enter the intestinal lumen, the worm recognizes foreign substances via C-type lectin domain proteins and activates xenobiotic detoxification programs through transmembrane transporters such as *pgp-5* and mediator components such as *mdt-15*. This tissue-specific response not only maintains metal homeostasis in the worm but also shapes the efficiency of initial pathogen colonization in the intestine. Some heavy metals, such as arsenic, cadmium, and hexavalent chromium, which are classified as Group 1 carcinogens by the International Agency for Research on Cancer (IARC), exhibit significant genotoxicity. In addition to causing indirect DNA oxidative damage through ROS pathways, they can directly block key defense systems such as nucleotide excision repair. For instance, arsenic inhibits DNA ligase activity, impairs damage repair, increases the frequency of gene mutations and chromosomal aberrations, thereby exacerbating genomic instability and ultimately inducing malignant transformation of cells into tumors (Figure 1 panel B). The table below will systematically summarize the environmental sources of key heavy metals and their core toxicological mechanisms in hosts (Table 1), laying the groundwork for subsequent discussion on how bacteria co-evolve specific resistance mechanisms and reshape their virulence characteristics in response to these toxic effects.

## 3. Molecular and Regulatory Effects of Heavy Metals on Bacteria

### 3.1. Sensing the Stress: Two-Component Systems (TCS) and Signaling Scaffolds

The precise pathogenicity of bacterial pathogens fundamentally relies on an evolutionarily developed, multidimensional molecular weapon system. The various virulence effectors integrated within this system exhibit spatiotemporal synergistic interactions that transcend individual functions during complex infection lifecycles [15]. For instance, the three-dimensional structure of the biofilm (co-constructed by the adhesin array, outer membrane protein components, and extracellular polysaccharide matrix) provides the material foundation for bacteria to recognize host targets and achieve stable initial colonization. This is complemented by the intervention of exotoxin proteins with targeted destructive capabilities and specialized secretion systems capable of precisely delivering effector molecules to dismantle the host’s innate immune defense barriers. This stepwise functional division, strictly aligned with the progression of infection, fundamentally reveals the ultimate evolutionary adaptation strategy shaped by pathogens under prolonged, stringent selection pressures within the host microenvironment [16].

Following successful initial attachment and establishment within specific host tissue microenvironments, pathogenic bacteria promptly shift to an aggressive invasion mode. Leveraging multiple sophisticated secretion systems (including Type I to VI systems that traverse cell membranes) they directionally pump various viral antigens and biologically active effector proteins into target host cells [17]. Among these, the highly specialized Type III and Type IV secretion systems possess a unique capability: they construct molecular injection needles connecting the bacterial cytoplasm to the host cytoplasm, enabling direct and precise transmembrane delivery of effectors [18]. They play a pivotal role in profoundly disrupting host innate immune signaling pathways, forcibly remodeling cytoskeletal protein aggregation states, and hijacking inflammatory response regulatory circuits. This underpins a complex molecular regulatory network that assists bacteria in maintaining dynamic survival homeostasis while balancing the dual demands of countering intense host immune defenses and satisfying their own metabolic nutrient acquisition needs [19].

As the core communication hub enabling bacterial populations to perceive environmental fluctuations at the microscopic level and achieve coordinated behavior among individuals, the quorum sensing system relies on concentration gradients of specific chemical signaling molecules in the extracellular microenvironment to monitor and precisely define local population density thresholds in real time. It then synchronously initiates a series of collective physiological responses (including the transcriptional expression of virulence factor-encoding genes, the three-dimensional construction of extracellular polysaccharide biofilms, and the activation of multidrug resistance defense mechanisms) using these thresholds as molecular command sources [20]. External physical and chemical signals (such as dynamic oscillations in metal ion concentration gradients, abrupt shifts in redox potential, or metabolic stress induced by nutrient depletion) often profoundly reshape the functional output of the quorum sensing network through indirect yet efficient molecular interference [21]. This occurs via microscopic pathways like allosteric regulation of specific transcription factor conformations or chemical modification of upstream signal transduction cascades. This multidimensional signal integration mechanism, seamlessly coupling intricate heterogeneous environmental stimuli with endogenous bacterial gene regulatory networks, fundamentally constitutes the decisive biological foundation enabling pathogenic bacteria to sustain their infectivity and pathogenic potential within the rapidly changing and hostile host microenvironment [22].

### 3.2. Quorum Sensing Interference

The profound interactive relationship established between the host organism and invading pathogenic microorganisms vividly reflects an evolutionary arms race spanning vast temporal and spatial dimensions. The pathogen’s continuously expanding and iteratively upgraded molecular arsenal of virulence factors represents not only a direct adaptive biological response to the stringent survival barriers erected by the host immune system, but also the fundamental driving force behind high-frequency diversification mutations in bacterial virulence factor genes to achieve antigenic escape [23,24]. Simultaneously, this pressure compels deep restructuring of intracellular core signaling regulatory networks at the molecular conformation and expression logic levels to precisely adapt to novel immune defense patterns. This bidirectional, mutually beneficial coevolutionary feedback mechanism, rooted at the microscopic molecular level, simultaneously charts the dynamic evolutionary trajectory of pathogenic microorganisms achieving adaptive survival within highly antagonistic host microenvironments, while unilaterally propelling bacterial infection strategies toward heightened complexity and sophistication [25].

**Figure 2:**
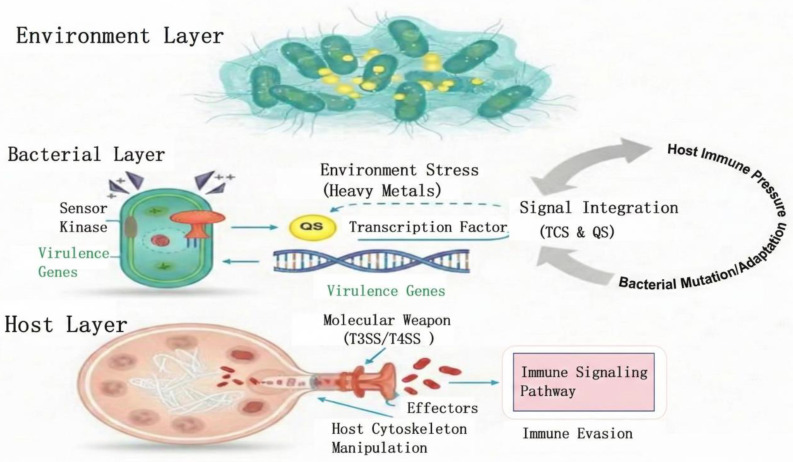
Multi-dimensional perspectives of bacterial pathogenic mechanisms: from environmental sensing to host interaction-synergistic regulatory networks. To clarify the visual transition from extracellular stress to intracellular response and host invasion, the symbols and formatting are defined as follows: **Arrow Shapes and Lines: Solid arrows** represent direct molecular pathways or confirmed biological processes (e.g., environmental stimuli activating sensor kinases). **Dashed-line arrows** signify indirect molecular interference or complex integration, such as the allosteric regulation of quorum sensing (QS) networks by environmental factors. **Large curved grey arrows** illustrate the bidirectional evolutionary “arms race” and feedback loops between bacterial adaptation and host immune pressure. **Color-Coding and Features:** Red structural components (e.g., the T3SS/T4SS apparatus and secreted effectors) highlight the “aggressive invasion mode” and the precise delivery of molecular weapons into the host. **Green text** (e.g., Virulence Genes) identifies the core genetic reservoir and transcriptional output synchronized by population density thresholds. **Pink-boxed text** denotes host-specific immune signaling pathways targeted for dismantling or hijacking. **Symbols:** Purple triangles and grey spheres represent environmental stressors (e.g., heavy metal ions). **The DNA helix and QS nodes** symbolize the core communication hubs enabling the spatiotemporal optimization of virulence gene expression.

The realization of bacteria’s precise pathogenic efficacy fundamentally relies on the highly synergistic coupling between diverse virulence effector clusters and underlying gene expression regulatory networks. The academic distinction between macro-level pathogenic potential and micro-level virulence intensity not only dissects the multidimensional structural characteristics of bacterial infectivity but also, through detailed classification of virulence factor functional attributes, fully reconstructs the pathogen’s entire chain of infection strategies ranging from breaching host physical barriers to evading immune clearance [26,27]. The entire molecular machinery of pathogenicity operates under the stringent oversight of higher-order global systems, such as two-component signal transduction and quorum sensing, ensuring the spatiotemporal optimization of virulence gene transcription. Furthermore, intense physicochemical stressors in the external environment (particularly heavy metal ion exposure) not only challenge bacterial survival but also profoundly reshape the ultimate pathogenic phenotype by infiltrating and heterogenizing these core signaling networks. This mechanism of environmental factor-driven adaptive drift in microscopic regulatory networks forms the theoretical foundation and logical starting point for exploring how heavy metals directionally regulate bacterial virulence evolution trajectories [28] (Figure 2).

## 4. Upregulation of Virulence Determinants and Co-Selection of Antibiotic Resistance

### 4.1. Enhanced Virulence and Secretion Systems

Heavy metals regulate bacterial virulence through multi-level mechanisms, primarily manifested as interference with signaling networks, induction of biofilm formation, and interactions between oxidative stress and virulence regulation.

Heavy metals regulate virulence by disrupting quorum sensing and two-component signal transduction systems. This direct activation mechanism drives downstream gene expression and reshapes collective behavior. The *Pseudomonas* ColRS system maintains membrane integrity and adaptability under multiple metal stresses (zinc, iron, chromium, and manganese) [29], while the *Escherichia coli* (*E. coli*) CusS-CusR system activates *cusCFBA* genes to maintain homeostasis by sensing copper and silver concentrations [30]. Beyond these, the Rcs (regulator of capsule synthesis) phosphorelay system, a complex multi-component signaling pathway, acts as a crucial sensor for cell envelope stress. It integrates environmental cues to co-regulate the expression of various virulence factors and capsule polysaccharide synthesis, thereby modulating bacterial environmental fitness and pathogenic potential. Such signaling pathways, widely involved in chemotaxis and metabolism, establish the foundation for bacterial infection in complex habitats [31].

In worm infection models, the host restricts pathogen proliferation through a defense mechanism known as nutritional immunity, whereby essential metal ions in the intestinal lumen (such as iron, Fe^2+^, and zinc, Zn^2+^) are chelated to deprive bacteria of the resources required for survival [32]. However, environmental exposure to heavy metals (e.g., Cd^2+^ or Co^2+^) can severely compromise this protective barrier: owing to their physicochemical similarity, these ions can competitively occupy binding sites on host metal-binding proteins or, via molecular mimicry, generate false iron signals [33]. Such disruption produces two detrimental consequences—on the bacterial side, fluctuations in copper or cobalt mislead bacterial sensing systems into perceiving an “iron-starved” state, thereby driving excessive synthesis and secretion of copper-transport–related toxins (e.g., aeruginonin); on the host side, when metal homeostasis regulators (such as MTF-1 and the zinc homeostasis system) are occupied by exogenous metals, the host loses control over the intestinal microenvironment, accelerating pathogen colonization and ultimately triggering a lethal hypoxic response [34,35].

Heavy metal-mediated metabolic regulation induces extracellular polysaccharide secretion and alters contaminant bioavailability, thereby enhancing degradation efficiency [36]. Metal-sensing two-component systems based on this mechanism have evolved into potential antimicrobial targets, exemplified by the disruption of polymyxin resistance through inhibition of the PmrB kinase in *Acinetobacter baumannii* [37]. Beyond Gram-negative pathogens, heavy metal resistance in Staphylococcus species has been extensively documented, particularly involving the Staphylococcal Cassette Chromosome (SCC) elements. These mobile genetic elements often carry resistance determinants such as SCC-cad/cop/ars, which confer tolerance to cadmium, copper, and arsenic, respectively. The co-localization of these metal-resistance genes with virulence factors or antibiotic resistance genes (e.g., mecA) on SCC elements facilitates horizontal gene transfer, suggesting that environmental heavy metal pressure may inadvertently select for more virulent or multidrug-resistant staphylococcal strains [38].

Sublethal concentrations of heavy metals induce biofilm formation and elevate mutation rates, selecting for multidrug-resistant strains to enhance environmental adaptability [39]. Combined exposure to antibiotics and heavy metals further strengthens adhesion and structural stability in strains such as *Kluyvera cryocrescens* and *Serratia fonticola* [40].

### 4.2. Biofilm Architecture and EPS Transformation

Molecularly, cadmium, lead, nickel, and mercury induce bacteria to switch from planktonic growth modes. They construct defense barriers by adsorbing heavy metals via EPS. Metal stimulation transforms EPS from spherical particles into highly adsorbent planar particles [41,42]. Low-concentration cadmium synergistically increases polysaccharide proportions with montmorillonite, while C–OH and PO functional groups jointly participate in metal binding [43,44]. Biofilms utilize EPS to directly bind metals, mitigating toxicity while impeding antibiotic penetration and activating silenced genes to enhance resistance [45,46,47]. They modulate host immune responses from pro-inflammatory to anti-inflammatory and pro-fibrotic states, coupled with protease degradation of immune components, ultimately establishing bacterial long-term persistence [48,49].

### 4.3. Oxidative Stress as a Virulence Regulator

Heavy metals catalyze ROS production, triggering oxidative stress. This stress interacts with sulfhydryl groups and iron metabolism, altering membrane permeability and promoting ROS accumulation to regulate virulence factor expression [50]. For instance, copper enhances pyrroloquinoline quinone synthesis in *Pseudomonas aeruginosa*, while *Salmonella* activates iron uptake systems under oxidative stress, both demonstrating the coupling of metal toxicity with adaptive mechanisms [51]. The glutathione system in streptococci also synergistically participates in resisting oxidative stress and metal toxicity [52].

At the transcriptional level, OxyR and SoxR transcription factors synergistically regulate antioxidant and metabolic gene expression. OxyR modulates ROS detoxification enzymes and metabolic genes to influence pathogenicity in multiple strains, including *Paraburkholderia xenovorans* [53], while SoxR synchronously controls antioxidant and metabolic gene networks in *Streptomyces avermitilis* and *E. coli* [54,55].

Small regulatory RNAs rapidly respond to metal stress and oxidative stress through post-transcriptional mechanisms, playing pivotal roles in virulence regulation. For instance, sRNAs in *Streptococcus mutans* regulate metal transport and oxidative tolerance [56], sRNA21 in *Mycobacterium abscessus* promotes antioxidant enzyme synthesis, and sRNAs in *Rhodobacter sphaeroides* resist oxidative damage by regulating C1 metabolism [57]. Direct intervention of sRNAs on virulence factors is exemplified by *Brucella abortus* regulating MurF enzyme activity and *Erwinia amylovora* interacting with Hfq to mediate type III secretion and biofilm formation [58,59].

**Figure 3:**
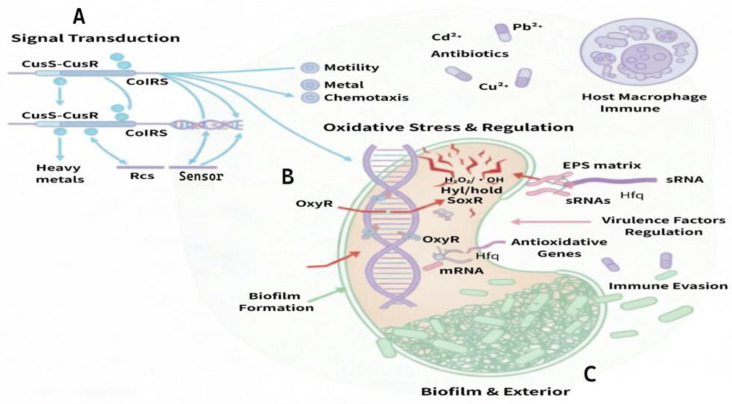
Multi-level regulation of bacterial virulence mediated by heavy metal. To facilitate the interpretation of the mechanistic model, the visual elements are defined as follows: **Sub-figure Scope:** (**A**) Metal-sensing signal transduction systems (e.g., CusS-CusR, ColRS, and Rcs phosphorelay) that modulate bacterial motility and chemotaxis; (**B**) Transcriptional regulation involving the coordination of OxyR/SoxR with sRNAs and Hfq chaperones; (**C**) Biofilm architecture and EPS transformation pathways under heavy metal stress. **Arrow Shapes and Colors: Solid arrows:** Represent direct molecular activation, signal transduction, or confirmed biochemical pathways (e.g., Cd^2+^ activating the Rcs system). **Light blue arrows:** Denote general signal transduction and metabolic flux within the cell (e.g., CusS-CusR and ColRS systems). **Red arrows:** Highlight the induction of oxidative stress and the production of reactive oxygen species (ROS). **Pink arrows:** Indicate the coordination and post-transcriptional regulation mediated by sRNAs and Hfq, as well as pathways related to host-pathogen interactions, including virulence factor regulation and immune evasion mechanisms. **Green arrows:** Mark the distinct physiological pathway leading to biofilm formation and environmental persistence.

In summary, the heavy metal-mediated two-component system, biofilm induction, and oxidative stress activation pathways coordinate collective behavior and virulence factor expression, forming a multi-level coupled regulatory network that establishes the molecular basis linking environmental pollution to infection risk (Figure 3).

## 5. Tripartite Interactions in the *C. elegans* Model

### 5.1. Model Advantages and Conserved Immunity

*C. elegans*, with its transparent body wall structure, provides researchers with a transparent window for real-time visualization of pathogen invasion trajectories and dynamic monitoring of host tissue pathological damage progression in vivo [60]. Its evolutionarily highly conserved innate immune system resembles that of mammals. For instance, the PMK-1/p38 MAPK signaling cascade precisely regulates downstream immune effector gene transcription by phosphorylating the transcription factor ATF-7—a molecular mechanism homologously mapped in the immune networks of higher mammals [61]. The complex defense network built upon this foundation integrates not only a detection system utilizing C-type lectin domain proteins to specifically recognize exogenous pathogen-associated molecular patterns, but also encompasses deep mechanisms where the nervous system modulates immune response intensity through G protein-coupled receptors like NPR-1 and OCTR-1 and neurotransmitter-mediated pathways [62]. The nematode’s ability to initiate specific transcriptional programs tailored to the type of invading pathogen, coupled with the functional homology between its Toll-like receptors, insulin-like growth factors, and other key signaling pathways and those of higher organisms, collectively establishes this species as a core reference for deciphering complex host pathogen interaction mechanisms [63].

The PMK-1/p38 and KGB-1/JNK-like MAPK cascades form an intricate intracellular signaling network through shared upstream regulatory elements. Under UV radiation-induced specific activation of the MOM-4/JKK-1 kinase, the former pathway not only initiates targeted transcription of immune effector genes via phosphorylation of the transcription factor ATF-7 but also synchronously activates downstream gene clusters of SKN-1/Nrf and DAF-16, thereby conferring potent tolerance to heavy metal toxicity such as cadmium [7,61]. The KGB-1 pathway, which forms a functionally complementary effect with it, not only extensively participates in reproductive development cycles, individual lifespan evolution, and protein synthesis regulation, but also serves as a critical defense barrier against high osmotic pressure fluctuations and heavy metal chemical stress, playing an irreplaceable role in nervous system development and axonal regeneration and repair [64]. The profound cross-regulatory relationship established between these two pathways across dimensions such as stress response, immune modulation, and life cycle determination enables KGB-1 to coordinate systemic stress responses through non-autonomous cellular mechanisms. It synergistically couples with PMK-1 to maintain the dynamic equilibrium of the apoptosis and autophagy pathways, ultimately jointly defining an organism’s developmental trajectory and adaptive resilience within complex and variable environments [65].

Under the dual environmental stresses of bacterial pathogen invasion and heavy metal ion chemical exposure, the adaptive upregulation of transcription levels for key transmembrane transporter family members like *pgp-5* profoundly reflects an intrinsic, tightly coupled mechanism between intracellular metal ion homeostasis maintenance networks and oxidative stress defense systems [66]. For instance, the *pmtA* metal efflux pump system in *Streptococcus pyogenes*, specifically regulated by the PerR factor (not only serves as a physical bridge for the interactive regulation of metal metabolism balance and redox status, but its overexpression-induced increase in AdcR-regulated gene expression further demonstrates the crucial role of transporters in responding to complex stresses). This molecular response paradigm at the prokaryotic level exhibits high functional correspondence with the high-expression response characteristics of the *pgp-5* gene in the aforementioned nematode model. This strongly suggests that homologous transporter gene clusters play a key regulatory role in maintaining the dynamic equilibrium of the hostpathogen environment interaction system.

Through a multidimensional experimental matrix combining transgenic nematode engineering strains, high-sensitivity fluorescent reporting systems, and survival analysis models, precise systematic assessment of the dynamic nonlinear coupling relationships among environmental metal ion concentration gradients, pathogen infection intensity, and host immune defense responses is achievable. The real-time in situ imaging capability of tissue-specific metal-sensing reporter genes for spatiotemporal distribution patterns of metallic elements within organisms, combined with the visual representation of defense signaling pathway activation states throughout the pathogen infection cycle via immune reporter genes, when deeply integrated with statistical analysis of individual survival rates, in vivo pathogen colonization load measurements, and RNA interference (RNAi)-mediated targeted gene silencing technology, this approach not only precisely reveals the deep molecular mechanisms underlying heavy metal chemical exposure, by reshaping core immune signaling networks to directionally alter host susceptibility pathways. Moreover, it offers profound insights into the intrinsic logical connections between cellular metal homeostasis maintenance systems and innate immune defense mechanisms from an evolutionary conservation perspective. Simultaneously, it provides a reliable experimental methodological basis for deciphering the complex regulatory effects of heavy metals on final infection outcomes and systematically evaluating the interaction patterns between environmental stressors and host defense mechanisms [67].

### 5.2. Sensory Modulation and Avoidance Behavior

Heavy metals increase the risk of infection in nematodes not only by weakening immune pathways but also by manipulating avoidance behavior. *C. elegans* relies on a highly developed sensory nervous system—mediated by G protein coupled receptors such as NPR-1 and OCTR-1—to detect chemical cues emitted by pathogens and to actively retreat from high-risk environments. Experimental evidence indicates that low-dose exposure to heavy metals (e.g., Pb^2+^ or Cd^2+^) exerts neurotoxic effects that disrupt sensory neuronal output, leading to neural “silencing” or biased signaling [68]. As a result, when confronted with highly pathogenic bacteria such as Pseudomonas aeruginosa, the normally protective avoidance response is delayed or lost: worms no longer move away from bacterial colonies, increasing physical contact and ingestion and thereby directly elevating infection probability. This behavioral shift is regulated through cross-tissue control by the KGB-1/JNK pathway, highlighting how heavy metals can undermine nematode survival strategies from within via the neuro immune axis [69,70].

### 5.3. Tripartite Interaction: Pathogen Host Metal Dynamics

Using the *C. elegans* model, the study revealed the dynamic relationship among heavy metals, pathogens, and hosts, confirming the pivotal role of heavy metals as virulence modulators.

At the pathogen level, heavy metal exposure significantly alters bacterial pathogenicity. For example, the enhanced virulence mechanism of *Pseudomonas aeruginosa* is closely linked to heavy metal interference with iron homeostasis. Its iron-transporting toxin, aeruginonin, induces lethal hypoxia responses in infected nematodes. Exogenous metals like cadmium and cobalt disrupt metabolic sensing by competing for iron-binding sites or mimicking iron signaling, thereby regulating toxin synthesis and secretion [71,72,73]. Fluctuations in iron homeostasis affect biofilm formation and antibiotic resistance. By regulating iron uptake metabolism via small RNAs (e.g., *prrF*), they enhance antimicrobial substance production to boost overall competitiveness and virulence [74,75,76]. Metal exposure also elevates ROS levels, upregulates iron uptake systems like *fep* and *fhu* alongside virulence operons such as *las* and *rhl*, and coordinates biofilm formation and virulence expression through quorum sensing systems [77].

*Staphylococcus aureus* exhibits similar patterns: heavy metals disrupt host nutritional-immune balance, activate tolerance and virulence factor expression, and enhance biofilm formation. Metal stimulation upregulates the *icaADBC* gene cluster, promoting synthesis of the polysaccharide intercellular adhesive substance (SICA) to enhance adhesion and immune evasion [78,79]. Oxidative stress or specific metal combinations suppress *icaADBC* expression, thereby reducing biofilm formation and infection risk [80,81].

### 5.4. Remodeling of Host Defense Responses

At the host level, shared defense modules activated by heavy metal exposure and pathogen infection are exemplified by the *C. elegans pgp-5* gene, which is upregulated under dual stress to execute detoxification and immune functions [82]. The PMK-1/p38 and KGB-1/JNK-like MAPK signaling pathways mediate integrated stress responses via shared transcription factors. PMK-1 phosphorylates ATF-7 to activate immune genes, while KGB-1 regulates heavy metal and protein folding stress genes through FOS-1. Additionally, KGB-1 collaborates with MTF-1 to maintain zinc homeostasis and antioxidant responses, and is associated with NF-κB signaling [83,84]. Additionally, the heat shock response collaborates with WRKY/RSMYB transcription factors to coordinate detoxification, antioxidant, and defense gene expression, collectively establishing a multi-tiered defense network [85].

### 5.5. General Patterns of Infection

**Figure 4:**
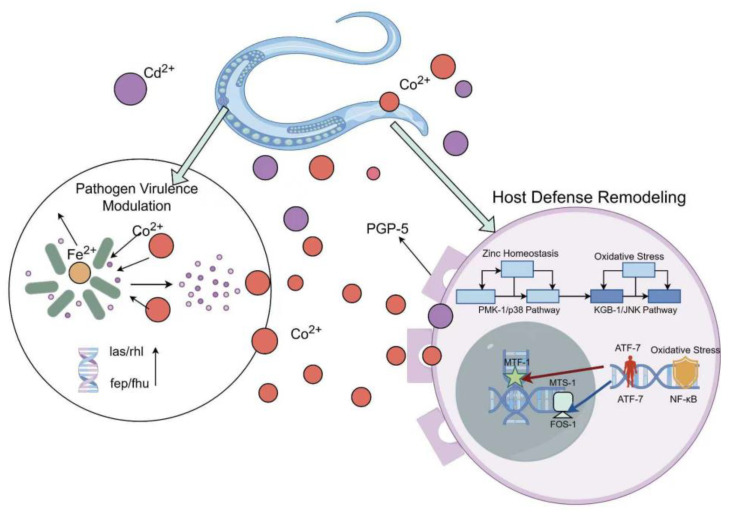
The performance of tripartite interactions in the *C. elegans* model. This schematic illustrates the dynamic coupling between heavy metal exposure, pathogen virulence modulation, and host defense remodeling. **Arrows and Signaling: Large hollow arrows:** Represent the systemic impact of heavy metals on both the pathogen (left) and the host (right). **Solid black/blue arrows:** Indicate direct biochemical signaling pathways or gene activation (e.g., Co^2+^ inducing iron-starved sensing). **Red arrows:** Highlight stress-induced destructive pathways, such as the activation of MTF-1 and subsequent transcriptional remodeling. **Circles and Symbols: Small colored circles:** Represent different heavy metal ions in the environment; purple circles denote Cd^2+^, while red circles denote Co^2+^. **Tan circle (Fe^2+^):** Represents the essential iron pool, which is subject to competition and mimicry by exogenous metals. **Green capsules:** Represent bacterial pathogens within the host or environment. **Special Geometric Shapes: Yellow Star (on DNA):** Signifies the MTF-1 (Metal-responsive Transcription Factor) activation site, representing a core node for sensing zinc and heavy metal homeostasis. **Grey Triangle/Trapezoid (on DNA):** Represents the FOS-1 transcription factor docking site, specifically involved in the KGB-1/JNK-mediated stress response and axonal repair mechanisms. **Shield Icon:** Represents the NF-κB signaling and general innate immune defense barriers. **Host Components:** The purple semi-circles on the cell membrane represent the PGP-5 efflux pump system, which is adaptively upregulated to maintain intracellular metal balance and mitigate oxidative stress.

The role of heavy metals in infection is not simply linear but forms a dynamic equilibrium through dual regulation by pathogen virulence and host defense. Low doses can induce host adaptive defense, such as upregulating the antimicrobial peptide gene *lys-7* to enhance innate immunity. Conversely, high doses or prolonged exposure disrupt homeostasis by enhancing bacterial virulence, accelerating host mortality while exacerbating oxidative stress, inflammation, and apoptosis [86,87,88,89]. These dose- and type-dependent effects (influenced by specific metal properties; see Table 1) determine the diversity of host defense responses and infection outcomes, revealing heavy metals’ pivotal ecological role in the interaction between environmental pollution and infectious disease risk (Figure 4).

## 6. Synthesis and Future Outlook

### 6.1. Environmental Signal Regulation Model

Existing research proposes the “environmental signal regulation model” as a conceptual model, which explains how sublethal concentrations of heavy metals act as environmental signals. Through quorum sensing and two-component signaling systems, these signals integrate information, activate virulence genes, trigger phenotypic shifts, and induce pre-activation in pathogens. This process occurs before host invasion, endowing pathogens with enhanced adhesion, drug resistance, and virulence characteristics [90]. The *Pseudomonas aeruginosa* GacS/GacA and LadS/PA0034 systems, along with the *Staphylococcus aureus* SaeRS system, perceive metal metabolic signals to regulate biofilm virulence and immune evasion. The *Listeria* CopRS system utilizes copper sensing to mediate tolerance and gene expression. Host-induced metal stress activates the glutathione (GSH) redox system (Trx) and the MDT-15/MED15 detoxification mechanism, which exhibit complex cross-regulation with the MAPK antimicrobial peptide immune pathway. Such nodes, balancing defensive coordination and pathogen exploitation risks, determine the final infection outcome [65].

### 6.2. Research Gaps and Future Directions

Existing studies focusing on single metals or short-term exposures fail to capture the reality of widespread low-dose mixed-metal exposure in the environment. Future research should shift toward investigating synergistic antagonistic effects of mixed metals, chronic exposure-induced genetic and epigenetic changes, and even transgenerational immune susceptibility. Heavy metals regulate host gene expression and developmental outcomes by manipulating DNA methylation and histone modifications, thereby reshaping offspring immune characteristics. Their disruption of the host microbiome alters metabolites like short-chain fatty acids and microbial community structure, weakening host defenses and facilitating pathogen invasion. Unraveling the “host microbiome environment” tripartite interaction mechanism will ultimately clarify the molecular basis of infection risk and guide intervention strategy development.

Current understanding of the heavy metal bacterial virulence host tripartite interaction remains largely descriptive. Our future research will therefore focus on developing integrated predictive relationships linking metal dosage, virulence output, and host survival using *C. elegans* model data [71], to support the reassessment of public health exposure thresholds.

### 6.3. Insights from Nematodes to Public Health

The *C. elegans* model confirms that environmental pollution exacerbates infection risk through an indirect pathway by enhancing microbial virulence. Low-dose heavy metal exposure induces host adaptive defenses like upregulation of antimicrobial peptides, while high-dose chronic exposure accelerates host decline by disrupting homeostasis through amplified pathogen virulence. Fluctuations in environmental conditions encompassing exogenous factors like temperature, pH, minerals, nanoparticles, and sweeteners profoundly influence microbial pathogenic phenotypes. This necessitates incorporating pollutant-induced microbial virulence enhancement indicators into environmental risk assessment systems. Simultaneously, adopting a One Health perspective to integrate human animal environment interdependencies will strengthen the scientific foundation for public health and environmental management.

While *C. elegans* provides a high-throughput platform for deciphering the fundamental “tripartite interaction,” the translational gap between this invertebrate model and human clinical outcomes must be acknowledged. The primary limitation lies in the absence of an adaptive immune system; unlike humans, *C. elegans* lacks specialized immune cells (e.g., T-cells, B-cells) and antibody-mediated memory, relying exclusively on an evolutionarily conserved innate immune architecture. Consequently, the model can accurately reflect heavy metal-induced shifts in primary defense barriers—such as intestinal mucosal integrity and the PMK-1/p38 MAPK-regulated antimicrobial peptide response—but it cannot simulate the long-term impact of pollutants on vaccine efficacy or the maturation of systemic immune memory. For human risk assessment, *C. elegans* serves as a “first-tier” sentinel system to identify the molecular potential for heavy metal-driven virulence enhancement and initial host susceptibility; however, these findings must be integrated with higher vertebrate data to fully characterize the complex clinical pathology of environmental pollutants in the Anthropocene.

## 7. Conclusions

Heavy metals (such as As, Cd, Pb, etc.), with their sources and toxicity detailed in Table 1, have transcended the realm of traditional chemical toxins, evolving into key signaling factors that actively regulate microbial behavior and host pathogen interactions within environmental ecological networks. By disrupting bacterial regulatory networks, they reshape host defense systems and profoundly intervene in the occurrence and transmission of infectious diseases. The *Caenorhabditis elegans* model, combining simplicity with physiological relevance, has elucidated the complex “environment microbe host” tripartite interaction mechanism. It also lays the scientific foundation for reforming public health risk assessment systems. Facing this “invisible threat,” future interdisciplinary, multi-level integrated research will be crucial for achieving a deep understanding and effective response.

## Figures and Tables

**Table 1:** Summary of environmental sources, host toxicological mechanisms, and cross-species analogies.

Heavy Metal	Major Target Organs in Humans	Corresponding Tissues/Responses in *C. elegans*	Key Toxicity Mechanisms (Conserved)	References
**Arsenic (As)**	Skin, Respiratory system, Nervous system	Sensory neurons (triggering avoidance behavior) and Intestinal cells (SKN-1/Nrf-mediated defense)	ROS production; enzyme inactivation via thiol binding; DNA repair interference.	[1,2,10]
**Cadmium (Cd)**	Kidneys, Bones, Respiratory system	Intestinal cells (upregulation of pgp-5 and mdt-15 detoxification)	Interferes with Ca/Zn homeostasis; protein misfolding; nephrotoxicity analog.	[1,2,5]
**Lead (Pb)**	Nervous system, Hematopoietic system	Nervous system (axonal regeneration stress via KGB-1) and Innate immunity (PMK-1/p38 signaling disruption)	Inhibits heme synthesis enzymes; disrupts Ca signaling; neurotoxicity.	[1,5]
**Copper (Cu)**	Liver, Nervous system, Kidneys	Intestinal cells (MTS-1/MTF-1 zinc/copper homeostasis)	Catalyzes Fenton reaction to produce ROS; damages DNA.	[1,2]
**Zinc (Zn)**	Gastrointestinal tract, Immune system	Intestinal barrier and PMK-1 pathway (modulating antimicrobial peptide expression)	Disrupts essential metal absorption; induces oxidative stress at high doses.	[1,4]

## Data Availability

This is a review article, and no new experimental data were generated. All data cited in this article have been referenced to their original sources, and readers may refer to the corresponding references for the original data.
